# Phase-Retrieval Algorithm for Hololens Resolution Analysis in a Sustainable Photopolymer

**DOI:** 10.3390/polym17202732

**Published:** 2025-10-11

**Authors:** Tomás Lloret, Víctor Navarro-Fuster, Marta Morales-Vidal, Inmaculada Pascual

**Affiliations:** 1 Instituto Universitario de Física Aplicada a las Ciencias y las Tecnologías, Universidad de Alicante, Carretera San Vicente del Raspeig s/n, 03690 San Vicente del Raspeig, Spain; 2 Departamento de Óptica, Farmacología y Anatomía, Universidad de Alicante, Carretera San Vicente del Raspeig s/n, 03690 San Vicente del Raspeig, Spain; 3 Departamento de Física, Ingeniería de Sistemas y Teoría de la Señal, Universidad de Alicante, Carretera San Vicente del Raspeig s/n, 03690 San Vicente del Raspeig, Spain

**Keywords:** photopolymers, sustainability, volume holography, hololens, Gerchberg–Saxton, phase retrieval, resolution, imaging applications

## Abstract

In this paper, the iterative Gerchberg–Saxton (GS) phase-retrieval algorithm is employed to reconstruct the amplitude spread function (*ASF*) of hololenses (HLs) recorded on a sustainable PVA/acrylate-based photopolymer, Biophotopol, when working with a CCD sensor. The main objective of this work is to characterize the spatial resolution of HLs, which are key components in a wide range of optical systems, including augmented reality (AR) glasses, combined information displays, and holographic solar concentrators. The GS algorithm, known for its efficiency in phase retrieval without prior knowledge of the phase of the optical system, is used to reconstruct the *ASF*, which is critical for mitigating information loss during imaging. Spatial resolution is quantified by convolving the *ASF*s obtained with two resolution tests (objective and subjective) and analyzing the resulting image using a CCD sensor. The convolution process allows an accurate assessment of lens performance, highlighting the resolution limits of manufactured lenses. The results show that the iterative GS algorithm provides a reliable method to improve image quality by recovering phase and amplitude information that might otherwise be lost, especially when using CCD or CMOS sensors. In addition, the recorded hololenses exhibit a spatial resolution of 8.9 lp/mm when evaluated with the objective Siemens star chart, and 30 cycles/degree when evaluated with the subjective Random E visual acuity test, underscoring the ability of Biophotopol-based HLs to meet the performance requirements of advanced optical applications. This work contributes to the development of sustainable high-resolution holographic lenses for modern imaging technologies, offering a promising alternative for future optical systems.

## 1. Introduction

Holography, a three-dimensional image reconstruction technique, is based on the wavefront reconstruction principle published by Dennis Gabor in 1948 [1,2]. Such was the positive impact of this technique that Gabor received the Nobel Prize in Physics in 1971 [3]. This interesting method allows information to be recorded on a photosensitive material and has undergone substantial advances thanks to improvements in holographic recording materials [4]. In recent years, technology has advanced at an incredible pace, and holography has been one of the best-adapted and -incorporated fields [5]. Among the applications of holographic technology, holographic optical elements (HOEs) show great promise. They are optical elements that can be designed and adapted to any field of interest and can act like conventional optical elements that work through refraction or reflection, but in this case the physical basis is the phenomenon of diffraction [6]. The Soviet physicist Yuri Denisyuk first proposed the idea of a holographic mirror as a HOE concept in 1962 [7]. This idea is the basis of reflection holography.

Among the wide variety of HOEs that can be designed and recorded holographically, hololenses (HLs) are particularly attractive elements. They are holograms that act as diffraction gratings with variable spatial frequencies and can converge and diverge light, like conventional optical lenses, but in this case through the phenomenon of diffraction [8,9,10]. HLs are now a fundamental component of optical imaging systems and are used primarily in head-mounted displays for virtual and augmented reality [11,12,13,14,15,16] or as non-imaging systems in light deflectors and concentrators [17,18,19,20]. In these applications, the optical and imaging quality of HLs is very important. For this purpose, some authors have studied the resolution of HLs using the modulation transfer function (MTF) [21], Fourier transform [22,23], or the study of some quality metrics [24,25]. The MTF does not provide complete information about the resolution of HLs, since it only provides information about the cutoff frequency in a region of the image. For this reason, a good method is to study the convolution of an object via a resolution test with the impulse response of the HLs. Previous work examined the difference between obtaining resolutions with a CCD sensor and with a Shack–Hartmann wavefront sensor [26,27]. The resolutions obtained with the CCD sensor using the convolution theorem were less reliable than those obtained with the Shack–Hartmann wavefront sensor, since some of the information was lost due to the way in which the impulse response was obtained in that case, which was by capturing the intensity distribution of the image focal point and convolving it with a USAF resolution test.

The main applications of HLs are in new recording materials, which can work in the most specific situations and applications. The fabrication of HOEs often involves the use of various materials, such as silver halide emulsion, dichromated gelatin, photoresistors, photorefractors, or photopolymers [28,29,30]. In 1969, Close et al. [4] first employed photopolymers as holographic optical components. Since then, a wide variety of photopolymer compounds have been developed for optical applications. This is mainly due to their adaptability in terms of composition and design, in addition to other interesting qualities such as self-processing capability, affordability, variable thickness, good dimensional stability, high energy sensitivity, sharp angular selectivity, wide dynamic range, and flexibility. The importance of photopolymers in this field is rapidly increasing. However, commonly used hydrophilic photopolymers also include gelatin binders, poly(vinyl alcohol), and similar monomers. The unfavorable features of these photopolymers include the toxicity of some of their constituents; for example, acrylamide has a high propensity to cause cancer [31]. Recent advances in photopolymers reduce this problem by using low-toxicity materials instead of conventional solvents to improve environmental compatibility, such as acrylate-based photopolymers. In addition, these materials also offer good recycling qualities for use as holographic recording materials in optical applications.

In this work, we have studied the potential use of the Gerchberg–Saxton (GS) iterative phase-recovery algorithm to reconstruct the impulse response (ASF) of negative asymmetric hololenses recorded on a sustainable acrylate-based photopolymer called Biophotopol, evaluated at 473 nm and 633 nm. Two resolution tests were used: a Siemens star chart to obtain objective measurements, and a Random E visual acuity test to obtain a more perceptual approximation, which is essential in applications of holographic optical elements to the field of augmented reality. The results showed that the use of this algorithm significantly improves the resolution measurement when working with CCD sensors.

## 2. Materials and Methods

### 2.1. Photopolymer Preparation

**Figure 1:**
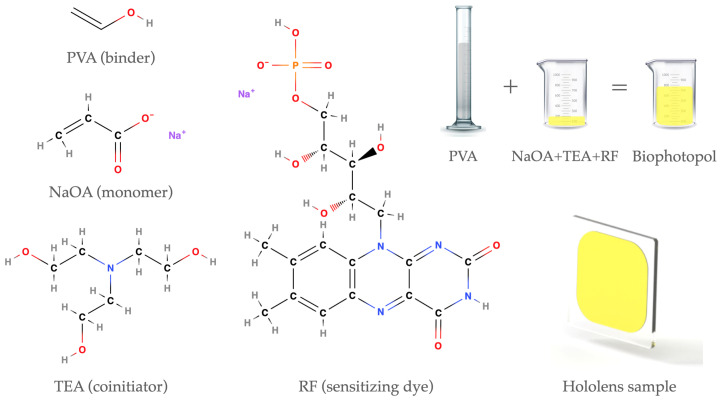
Schematic of the Biophotopol components’ chemical structures and example of a hololens. Binder: PVA (polyvinyl alcohol); monomer: NaOA (sodium acrylate); co-initiator and plasticizer: TEA (triethanolamine); and sensitizing dye: RF (riboflavin $5^{\prime }$-monophosphate sodium salt).

The holographic recording material used was an acrylate-based photopolymer called Biophotopol [32,33]. This is a hydrophilic sustainable material composed of a monomer in a binder, a co-initiator system, and a dye sensitizer. In previous works, the use of this material in different holographic applications was thoroughly studied [19,20,21,24,26]. The concentrations of Biophotopol components were optimized to obtain high diffraction efficiency (DE) in hololens storage (DE=90%). The final composition of Biophotopol was developed using sodium acrylate (NaOA) as a monomer (NaOA was generated in situ by a reaction of acrylic acid (HAO) with sodium hydroxide (NaOH) in a 1:1 ratio), water as the sole solvent, triethanolamine (TEA) as an initiator and plasticizer, riboflavin $5^{\prime }$-monophosphate sodium salt (RF) as a colorant, and polyvinyl alcohol (PVA) as a binder (Mw = 130.000 g/mol, degree of hydrolysis = 87.7%). Figure 1 shows a schematic of the chemical structures of the Biophotopol components, how they are mixed to form the final prepolymer solution, and an example of a hololens sample.

The optimized concentrations of the Biophotopol components, which allow the production of layers with maximum diffraction efficiency, are presented in Table 1. The prepolymer solution was deposited in 6.5 × 6.5 ${\mathrm{cm}}^{2}$ glass molds (previously subjected to a washing and drying process) by gravitational action. Subsequently, it was left in darkness in an incubator (Climacell 111, Labexchange, Burladingen, Germany) for a period of approximately 21 h, under controlled conditions of relative humidity (60 ± 5%) and temperature (20 ± 1 °C). In this process, the incubator functioned as a light-insulated environment, ensuring that the photopolymer layer was not affected by radiation. During the drying cycle, some of the water contained in the prepolymer solution evaporated, reaching hygrometric equilibrium with the surrounding environment inside the incubator. The physical thickness of the dried photopolymer layer was measured using a micrometer, obtaining a value of 150 ± 10 μm. The layers were then prepared for recording, which began immediately.

**Figure 2:**
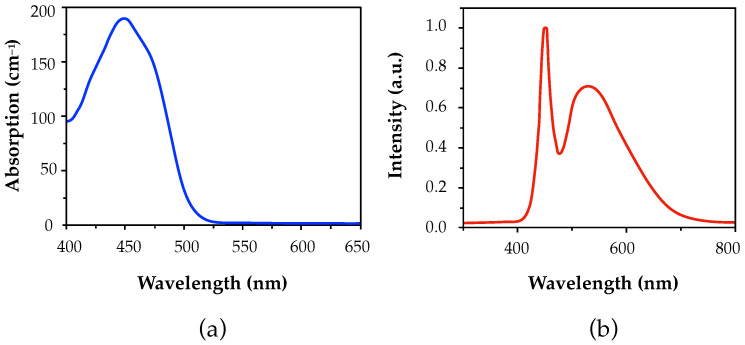
Characteristics of the dye absorption and the curing process. (**a**) RF absorption curve (blue line), and (**b**) LED lamp emission spectrum (red line).

Finally, once the hololenses were recorded, a curing or bleaching process was performed in order to finalize the chemical reactions of the photopolymerization process and make the material stable. The overall effect is that the photopolymer becomes more stable and less prone to undergoing additional chemical changes after the curing process. This process was performed with an LED lamp (13.5 W, 875 lm, 6500 K) for 10 min. Figure 2a shows the spectral sensitivity of the RF, where the curve represents the RF absorption at different wavelengths. It can be observed that Biophotopol has its maximum absorption peak around 450 nm. In addition, Figure 2b shows the emission spectrum of the LED lamp used during the curing process.

### 2.2. Holographic Experimental Setup for Writing and Reading

**Figure 3:**
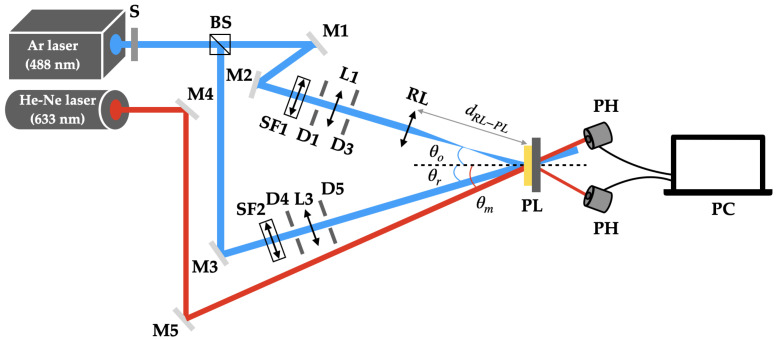
Experimental setup used for holographic lens recording and reconstruction. S: shutter; BS: beam-splitter; $M_{i}$: mirrors; $L_{i}$: lenses; RL: refractive lens; ${\mathrm{SF}}_{i}$: spatial filters; $D_{i}$: diaphragms; PL: photopolymer layer; PH: photodetectors; and PC: personal computer.

The holographic experimental setup used for recording hololenses is depicted in Figure 3. A beam from an Argon laser, operating at a wavelength of 488 nm (to which the material is responsive), was divided into two parts: a reference beam and an object beam, using a beam-splitter. Both beams were spatially filtered and collimated. The object beam then passed through a refractive lens (RL), forming a divergent beam. These two beams were combined at the photopolymer layer, hitting it at different angles of incidence ($\theta_{o}$ and $\theta_{r}$) relative to the normal of the photopolymer surface. The intensity ratio between the two beams was 1:1, with a total recording intensity of 3 mW/${\mathrm{cm}}^{2}$, which represents the sum of the individual beam intensities at the hologram plane. The exposure time was set to 20 s. Due to the interference between the beams, regions of constructive (bright) and destructive (dark) interference were created in the photopolymer layer. In the bright regions, a radical polymerization process occurred, leading to modulation of the refractive index.

Furthermore, it is important to note that since Biophotopol is not sensitive to wavelengths of 633 nm (as can be seen in Figure 2a), the recording process was monitored using a He-Ne laser, which achieved diffraction efficiencies of 90% after the curing process.

### 2.3. Experimental Setup to Analyze the Intensity Distribution in the Image Plane with a CCD Sensor

**Figure 4:**
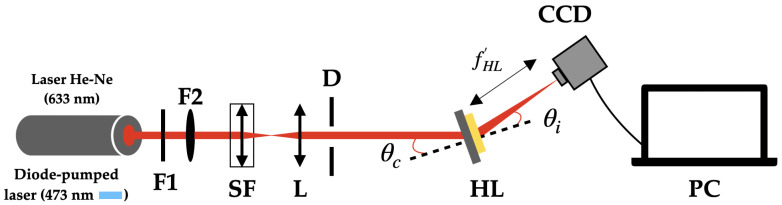
Experimental setup used to analyze the intensity distribution in the hololens image plane. $F_{i}$: neutral density filters; SF: spatial filter; L: lens; D: diaphragm; HL: holographic lens; CCD: charge-coupled device; and PC: personal computer.

The experimental setup used to analyze the intensity distribution in the image focal plane can be seen in Figure 4. The reconstruction of the HLs was performed with two wavelengths: 473 nm, which is close to the recording wavelength, and 633 nm, which is totally different. The CCD sensor was placed at the position of the image focal point of the holographic lens and formed an angle of $\theta_{i}$ with respect to the normal to the lens. The focal length and image angle were calculated using Equations (1) and (2), respectively, where the subscripts o, r, and c refer to the object, reference, and reconstruction waves; *R* represents the distance from the wave to the hologram; and θ is the angle between the wave and the normal to the hologram [34,35]. Once the CCD sensor was placed in the correct position, the amount of light was attenuated using filters F1 and F2, so that the sensor was not saturated.

(1) $$\frac{1}{f_{HL}}=\mu \left(\frac{1}{R_{o}}-\frac{1}{R_{r}}\right)$$

(2) $$\sin\theta_{i}=\sin\theta_{c}+\mu \left(\sin\theta_{o}-\sin\theta_{r}\right)$$

## 3. Computing Framework

### 3.1. Convolution Theorem and Amplitude Spread Function

Mathematically, a complex extended object can be represented as a weighted sum of impulse functions [36]. The impulse response of the HL is the amplitude spread function, which can be independent of the object plane position, in which case it is called invariant under translations. In addition, if there is no distortion in the system, the coordinates of the image plane are linearly related to the coordinates of the object plane through the lateral magnification *M*. Therefore, the image of an extended object can be calculated as an overlay of weighted ASF through the following direct operation:

(3) $$I\left(x,y\right)=\int \int O\left(u,v\right)\cdot ASF\left(u-\frac{x}{M},v-\frac{y}{M}\right)dudv$$

where O(u,v) and I(x,y) represent the object and the image, respectively. This integral is called convolution. Therefore, the image of a complex object can be seen as a convolution of that object and the impulsive response of the system. The impulse response function describes the response of an imaging system to an object point and can be defined using amplitude or intensity. For an optical system working with coherent light, and whose pupil is circular, the impulse response is given as the amplitude and is known as ASF. On the other hand, when the light source is incoherent, this function is called the point spread function (PSF), and represents the intensity distribution in the image plane. Table 2 shows the equations that describe ASF and PSF, where $J_{1}$ represents a first-order Bessel function.

In the case of an aberrated optical system, the impulse response can be defined as the Fourier transform of a complex function *P*, known as the generalized pupil function. It should be noted that the only effect of aberrations is to introduce phase distortions in the passband [37]. The ASF is defined as follows:

(4) $$ASF\left(x^{\prime },y^{\prime }\right)=A\cdot FT{\left[P(x_{p},y_{p})\right]}_{u=\frac{x^{’}}{\lambda s^{’}},v=\frac{y^{’}}{\lambda s^{’}}}$$

### 3.2. Gerchberg–Saxton (GS) Iterative Phase-Retrieval Algorithm

The Gerchberg–Saxton algorithm is an iterative algorithm that allows the phase of a wavefront to be determined from the intensity in two known planes [38,39,40,41,42,43]. Since both planes are related by the Fourier transform, with only one phase distribution, we obtain the phase distribution in the other plane. In this work, images from the Fourier plane (FP) and the focal image plane (IP) were used. The requirement that both planes must satisfy is as follows:

$$U_{FP}=F\left\{U_{IP}\right\}$$

(5) $$U_{IP}=F^{-1}\left\{U_{FP}\right\}$$

where the subscripts FP and IP denote Fourier and image planes, respectively, and *U* represents the complex field.

**Figure 5:**
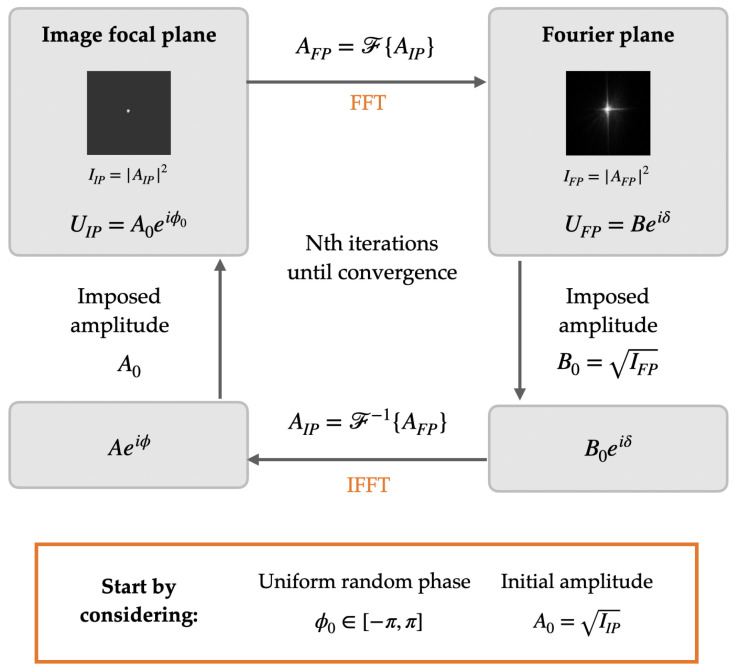
Flowchart of the GS phase-retrieval algorithm. Note: All variables are space-dependent, e.g., $A_{0}=A_{0}\left(x,y\right)$, although the (x,y) coordinates are omitted for simplicity.

Figure 5 shows a flowchart of the GS phase-retrieval algorithm. The restrictions applied in the algorithm are those described above, i.e., the amplitudes of the two images must converge. The first iteration of our algorithm begins with the propagation of the square root of the recorded intensity captured in the image focal plane (denoted as $A_{0}=\sqrt{I_{IP}}$). From there, we propagate to the Fourier plane using the fast Fourier transform (FFT) and analyze the field in that plane. As a constraint, we stipulate that the amplitude value in that field must be equal to the square root of the intensity captured in that plane ($\sqrt{I_{FP}}$), and we propagate back to the image plane using the inverse fast Fourier transform (IFFT) and again impose that the amplitude value in that plane be the same as the one we started with. Throughout this procedure, we preserve the phase information and let it evolve with the fast Fourier transform. We achieve convergence for N=30 iterations.

### 3.3. Resolution Using a Siemens Star Chart and a Visual Acuity Test

First, the maximum cutoff frequency of the hololenses was calculated to determine the maximum allowable frequency. The maximum attainable cutoff frequency corresponds to the diffraction-limited performance of an optical system. For a system operating under coherent illumination, this limit is given by

(6) $$F_{cut}=\frac{D}{2\lambda }\frac{1}{f_{HL}}\;\left(\mathrm{lp}/\mathrm{mm}\right)$$

where λ denotes the operating wavelength (473 nm and 633 nm), *D* is the aperture diameter of the hololens (D=12 mm), and $f_{HL}$ represents its focal length. The holographic lens provides a focal length of $f_{HL}=93$ mm at λ=473 nm, and $f_{HL}=70$ mm at λ=633 nm, respectively.

The hololens resolution was studied using two different methods: an objective method using the Siemens star chart test, and a subjective method using a visual acuity test.

First, a Siemens star chart was used to study the hololens resolution via an objective approach. This test represents *N* black and white sectors. All lines are concentrated in the center, where, as a consequence of the limited resolution, a gray circle appears. The resolution is mathematically defined as

(7) $$Resolution=\frac{N}{2\pi R\Delta x}$$

where *N* is the number of black and white sectors, *R* is the radius of the critical circle (gray circle), and Δx is the CCD sensor pixel size (in this work Δx=4.65 μm). To obtain the resolution, the radius of the gray circle is measured in pixels, and knowing the pixel size, it is easy to calculate the radius in millimeters, so to calculate the resolution, it would be necessary to measure the distance *d* and use Equation (7).

Secondly, a visual acuity test was conducted to study resolution in a more perceptual sense, specifically the Random E Chart visual acuity (VA) test [44]. This test is essential because it provides insight into the user’s visual perception when looking through the HLs. The resolution value derived from this test reflects the level of detail that the lenses can resolve. By converting the VA results into resolution measured in cycles per degree (c/deg), it is possible to simulate and quantify the expected visual experience when using the lenses. This approach provides a clear understanding of how much detail the hololenses can resolve and how the user will perceive them in terms of visual sharpness and clarity. In this way, the simulation with the VA test provides valuable information about the visual perception through the lenses, ensuring that their resolution is appropriate for an optimal augmented reality experience. Table A1 shows the relationship between VA and its corresponding spatial resolution, expressed in c/deg (see Appendix A).

## 4. Results

**Figure 6:**
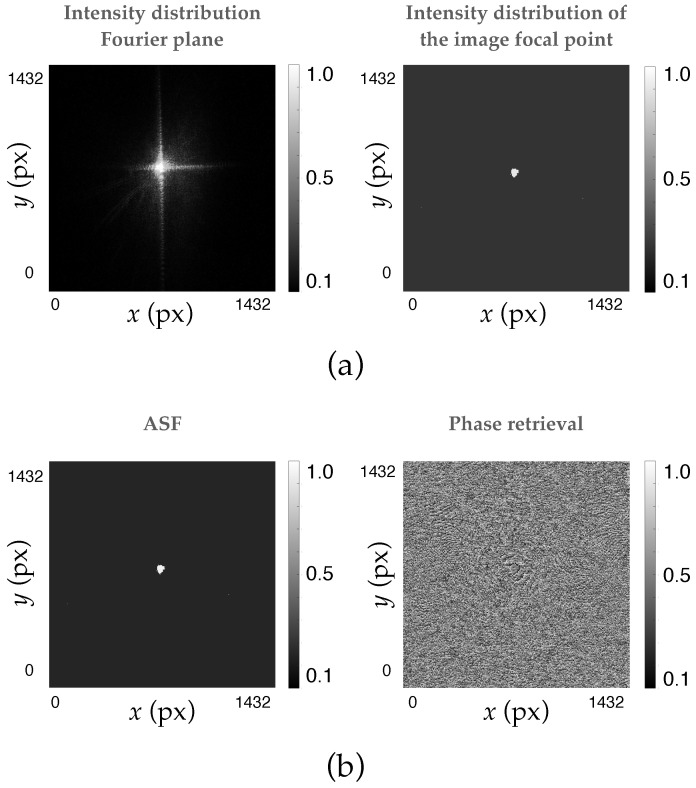
(**a**) Intensity images introduced in the algorithm, and (**b**) ASF and the phase reconstructed by the Gerchberg–Saxton method.

To avoid the problem of phase information loss when working with the CCD sensor, the Gerchberg–Saxton iterative phase-retrieval algorithm was used. To achieve this, two images of known intensity were taken: the intensity in the Fourier plane of the system and the intensity of the image focal point (where the intensity distribution was evaluated). The algorithm was applied and the number of iterations was optimized until convergence. In this specific case, the number chosen was N=30. Figure 6a shows the intensity images entered into the algorithm, and Figure 6b shows the ASF and the reconstructed phase.

### 4.1. Objective Evaluation of Optical Resolution Using the Siemens Star Chart

**Figure 7:**
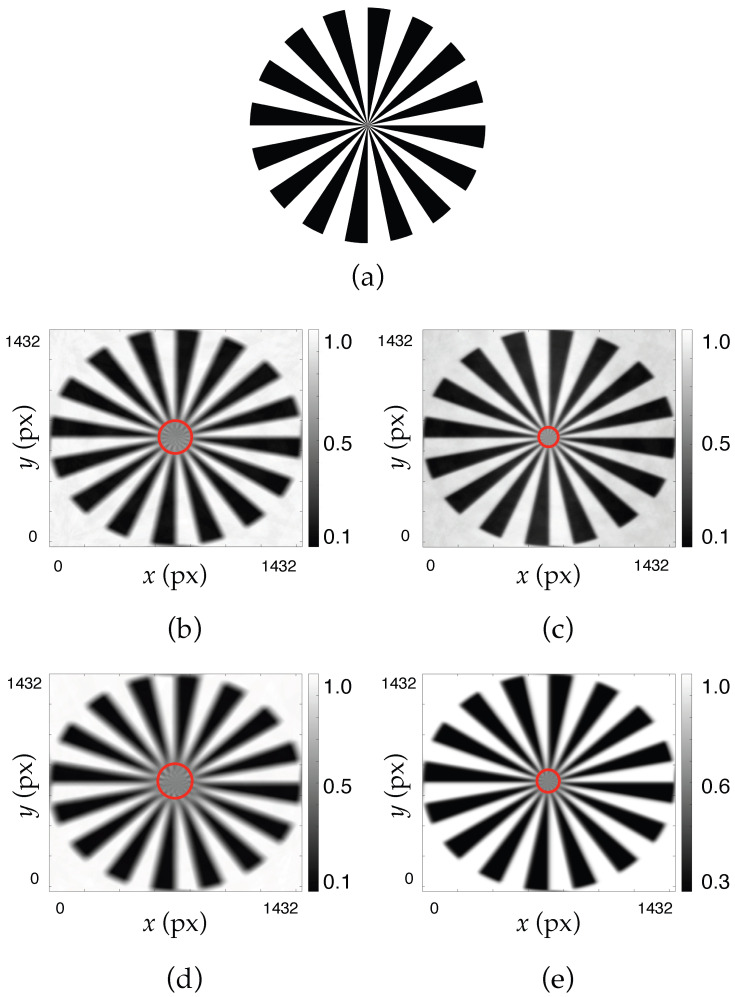
Simulated convolution of a Siemens star chart for negative asymmetric hololenses, with ASF obtained with the CCD sensor. (**a**) Siemens star chart test with N=16 black and white sectors (the test was designed in Matlab). Images obtained without using the GS algorithm (**b**,**d**) and using the GS algorithm (**c**,**e**); images evaluated at 473 nm (**b**,**c**) and and 633 nm (**d**,**e**).

To objectively evaluate the spatial resolution of the system, a Siemens star chart was used as a reference target under various experimental conditions (Figure 7a). The Siemens star is a well-established resolution test pattern that allows for the analysis of angular and radial resolution by taking advantage of its continuously varying spatial frequency from the periphery to the center. By analyzing the contrast of the concentric radii, especially where they begin to blur or merge, it is possible to estimate the holographic lens resolution.

In this study, Siemens star charts were evaluated at two wavelengths, 473 nm (Figure 7b,c) and 633 nm (Figure 7d,e), both with (Figure 7c,e) and without (Figure 7b,d) the application of the GS phase-recovery algorithm. Table 3 shows the resolution values, in lp/mm, obtained from Figure 7. It can be clearly seen that when the GS algorithm is applied, the resolution values increase for both wavelengths, 473 nm and 633 nm. The radii of the gray circle (*R*) are 112, 62, 121, and 68 pixels for Figure 7b, Figure 7c, Figure 7d and Figure 7e, respectively.

Without GS correction, both wavelengths showed a smoother contrast profile with earlier degradation, indicating more limited angular resolution. The image at 473 nm showed slightly better reference performance because at wavelengths closer to the recording wavelength, the impulse response exhibits fewer aberrations. However, after applying the GS algorithm, the contrast curves became noticeably sharper, and the resolution limit was extended closer to the center of the Siemens star, especially in the case of 633 nm. This suggests that the phase-recovery method effectively restores the higher spatial frequencies that were initially lost or distorted due to phase aberrations. Furthermore, by calculating the resolution in lp/mm, we can compare the results obtained in this work with those obtained in previous works [26,27] using other methods (resolution using the Hartmann-Shack wavefront sensor and resolution from a direct image of a physical USAF test). It can be seen that using the GS algorithm, the results are similar to those obtained with other methods such as a Hartmann–Shack wavefront sensor, suggesting that GS is a useful tool when working with CCD sensors.

### 4.2. Perceptual Evaluation of Angular Resolution Capacity

In order to complement the quantitative assessment of spatial resolution, a perceptual evaluation was conducted using the Random E chart visual acuity test under the same optical conditions. These charts, commonly used in visual acuity tests, offer a direct visual cue to assess the ability of the system to resolve fine detail as perceived by a human observer. By inspecting the legibility and sharpness of the alphanumeric characters at various scales, this approach provides intuitive feedback about the system’s effective angular resolution capacity, particularly relevant for real-world applications involving human visual perception.

**Figure 8:**
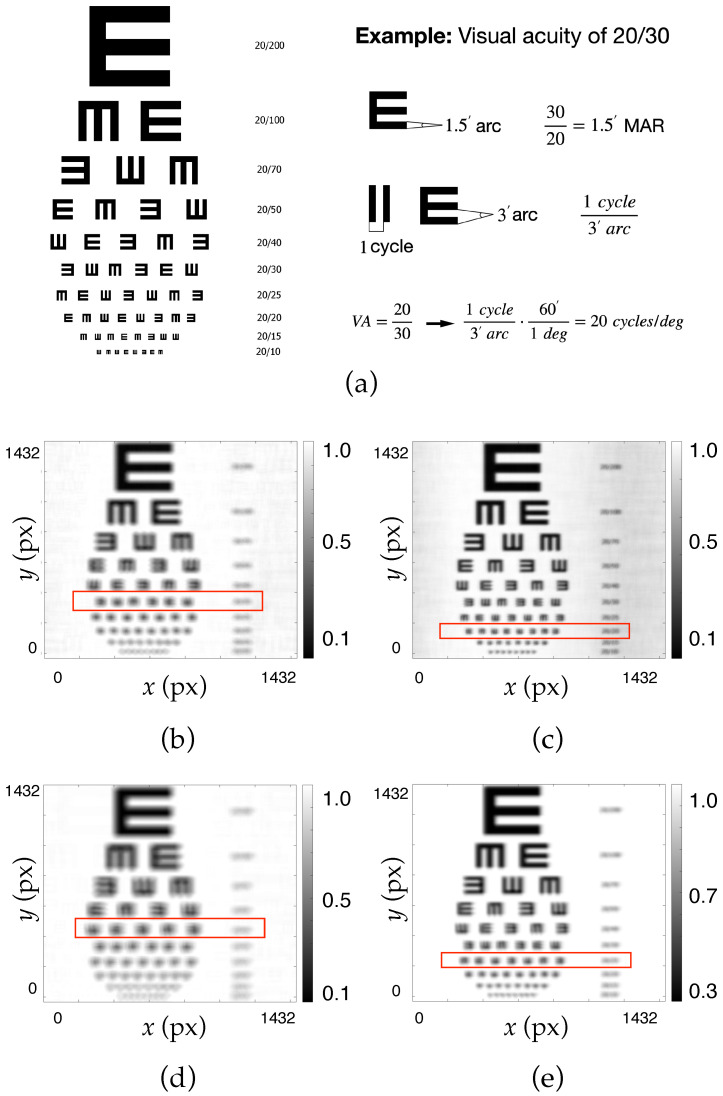
Simulated convolution of a Random E visual acuity test for negative asymmetric hololenses, with ASF obtained with the CCD sensor. (**a**) Original test and example of the conversion between VA = 20/30 and spatial resolution in cycles/degree. Images obtained without using the GS algorithm (**b**,**d**) and using the GS algorithm (**c**,**e**); images evaluated at 473 nm (**b**,**c**) and 633 nm (**d**,**e**).

Figure 8a shows the test used to obtain the resolution and an example of the conversion between VA = 20/30 and spatial resolution in cycles/degree. It also presents comparative results for evaluations at 473 nm (Figure 8b,c) and 633 nm (Figure 8d,e), both with (Figure 8c,e) and without (Figure 8b,d) the application of the GS-based phase-retrieval algorithm. In the uncorrected cases (without GS), the lower contrast and blurred edges make it difficult to distinguish finer letters, especially at 633 nm. This degradation is consistent with the system’s optical limitations and the increased sensitivity to shorter wavelengths. In contrast, the application of the GS algorithm leads to a significant perceptual improvement. The images reconstructed with GS reveal more legible characters, particularly in the smallest rows, indicating an enhancement in angular resolution. Notably, this improvement is not just numerical; it is visually discernible and thus more relevant to end-user experience.

Table 4 shows the spatial resolution results in c/deg for the images in Figure 8. It can be seen that for both the 473 nm and 633 nm evaluations, in both cases using the GS algorithm, the resolution is improved, reaching a maximum of 30 c/deg for the 473 nm evaluation, which is the wavelength closest to the recording wavelength.

This perceptual analysis is particularly critical when considering future applications in holographic near-eye displays for augmented reality, where visual clarity, legibility, and realism are paramount. The consistent performance gains observed across wavelengths demonstrate the robustness of the phase-retrieval method and its potential for deployment in vision-critical photonic systems.

The reconstructed ASFs and the phase maps obtained using the Gerchberg–Saxton phase-retrieval algorithm provided valuable information about the optical quality of the hololenses. The agreement between the objective Siemens star test and the subjective Random E chart test highlights the usefulness of the algorithm when working with CCD sensors. Furthermore, these results highlight the optical performance of the recording material. In this context, the use of Biophotopol as a recording material represents an important step towards the development of environmentally sustainable holographic systems. Unlike conventional photopolymers, which may contain toxic or volatile components, Biophotopol is based on a low-environmental-impact PVA–acrylate matrix, making it suitable for applications where user safety and ecological considerations are paramount, such as augmented reality (AR) devices. However, despite its promising optical performance, several aspects of the material require further optimization to ensure its long-term stability and reproducibility. The hydrophilic nature of the PVA matrix makes Biophotopol sensitive to environmental humidity, which can alter its diffraction efficiency due to phenomena such as swelling. Therefore, appropriate encapsulation or sealing strategies must be implemented to protect the holographic elements from humidity absorption. In addition, shrinkage must be precisely controlled to avoid deformation or spectral shifts in the engraved gratings. Finally, the concentration of the prepolymer components must be optimized based on the physical thickness of the engraving layer and the specific optical configuration of the device, as the balance between diffraction efficiency, dynamic range, and stability depends largely on the composition and geometry of the film. Addressing these challenges will be essential to fully exploit the potential of Biophotopol as a sustainable alternative for high-performance holographic optical elements.

## 5. Conclusions

In summary, the Gerchberg–Saxton iterative phase-recovery algorithm was optimized for use in holographic applications. Hololenses were fabricated using a sustainable acrylate-based photopolymer (Biophotopol), which was based on a PVA–acrylate matrix. This material demonstrated excellent optical performance and resolution, positioning it as a strong candidate for the development of holographic systems aimed at augmented reality applications. The amplitude impulse response was reconstructed from the intensity distribution obtained from the CCD sensor, and the resolution of different hololenses was obtained through convolution simulated imaging. Thanks to the choice of a sustainable photopolymer, HLs can be used in augmented reality systems without toxicity risks. This work demonstrates that the GS algorithm is a useful computational tool in the field of analog holography, since it allows us to reconstruct the impulse response of a system working with coherent light and with sensors that only capture intensity.

## Figures and Tables

**Table 1:** Biophotopol prepolymer composition quantities.

PVA (wt/V %)	NaAO (M)	TEA (M)	RF (M)
13.5	0.39	9.0 $\times \;10^{-3}$	1.0 $\times \;10^{-3}$

**Table 2:** Summary with the equations that describe ASF and PSF.

**Amplitude**	$P\left(u,v\right)=2\left(\frac{J_{1}\left(2\pi \sqrt{u^{2}+v^{2}}R_{ps}\right)}{2\pi \sqrt{u^{2}+v^{2}}R_{ps}}\right)$
**Intensity**	$I\left(u,v\right)={\left|2\frac{J_{1}\left(2\pi \sqrt{u^{2}+v^{2}}R_{ps}\right)}{2\pi \sqrt{u^{2}+v^{2}}R_{ps}}\right|}^{2}$

**Table 3:** Resolution values using the Siemens star chart.

λ (nm)	$F_{\mathrm{cut}}$ (lp/mm)	Resolution Before GS (lp/mm)	Resolution After GS (lp/mm)
473	67.7	4.9	8.8
633	68.2	4.5	8.1

**Table 4:** Resolution values obtained using the visual acuity test.

λ (nm)	$F_{\mathrm{cut}}$ (c/deg)	Resolution Before GS (c/deg)	Resolution After GS (c/deg)
473	110.7	20	30
633	82.7	15	24

## Data Availability

Data are contained within the article.

## Abbreviations

The following abbreviations are used in this manuscript:

HL	Hololens
HOE	Holographic optical element
PVA	Polyvinyl alcohol
NaOA	Sodium acrylate
TEA	Triethanolamine
RF	Riboflavin $5^{\prime }$-monophosphate sodium salt
GS	Gerchberg–Saxton iterative phase-retrieval algorithm
CCD	Charge-coupled device
CMOS	Complementary metal oxide semiconductor
ASF	Amplitude spread function
PSF	Point spread function
MTF	Modulation transfer function
VA	Visual acuity

## Appendix A. Relationship Between VA and Resolution for the Random E Chart Visual Acuity Test

Table A1 shows the relationship between visual acuity and spatial resolution in cycles/degree for the Random E chart visual acuity test.

**Table A1:** Conversion between visual acuity and spatial resolution.

Visual Acuity (Snellen Fraction)	Spatial Resolution (c/deg)
20/10	60
20/15	40
20/20	30
20/25	24
20/30	20
20/40	15
20/50	12
20/70	8.57
20/100	6
20/200	3

## References

[1] Gabor D. (1948). A New Microscopi Prinnciple. Nature.

[2] Gabor D. (1951). Microscopy by Reconstructed Wave Fronts: II. Proc. Phys. Soc. Sect. B.

[3] Gabor D. (1971). Holography. 1948–1971. Nobel Lecture, The Nobel Foundation. https://www.nobelprize.org/prizes/physics/1971/gabor/lecture/.

[4] Close D., Jacobson A., Margerum J., Brault R., McClung F. (1969). Hologram recording on photopolymer materials. Appl. Phys. Lett..

[5] Sheridan J.T., Kostuk R.K., Gil A.F., Wang Y., Lu W., Zhong H., Tomita Y., Neipp C., Francés J., Pascual I. (2020). Roadmap on holography. J. Opt..

[6] Schwar M., Pandya T., Weinberg F. (1967). Point holograms as optical elements. Nature.

[7] Denisyuk Y.N. (1962). Photographic reconstruction of the optical properties of an object in its own scattered radiation field. Sov. Phys. Dokl..

[8] Kostuk R., Russo J., Zhang D., Castro J., Vorndran S. (2016). Holographic Applications in Solar-Energy-Conversion Processes.

[9] Hariharan P. (2002). Basics of Holography.

[10] Syms R.R.A. (1990). Practical Volume Holography.

[11] Kress B., Cummings W. (2017). 11-1: Invited Paper: Towards the Ultimate Mixed Reality Experience: HoloLens Display Architecture Choices. SID Symp. Dig. Tech. Pap..

[12] Tseng E., Kuo G., Baek S.H., Matsuda N., Maimone A., Schiffers F., Chakravarthy P., Fu Q., Heidrich W., Lanman D. (2024). Neural étendue expander for ultra-wide-angle high-fidelity holographic display. Nat. Commun..

[13] Lin W.K., Matoba O., Lin B.S., Su W.C. (2018). Astigmatism and deformation correction for a holographic head-mounted display with a wedge-shaped holographic waveguide. Appl. Opt..

[14] Li G., Lee D., Jeong Y., Cho J., Lee B. (2016). Holographic display for see-through augmented reality using mirror-lens holographic optical element. Opt. Lett..

[15] Guo H., Xie Y., Lu D., Liao Y., Zhou X., Li Z., Peng H., Xie X. (2025). Reliable Holographic Plastic for Augmented Reality Display. Adv. Opt. Mater..

[16] Kaczorowski A. (2025). Holography and Its Applications in Augmented Reality.

[17] Haegel N., Verlinden P., Victoria M., Altermatt P., Atwater H., Barnes T., Case C., De Wolf S., Deline C., Dharmrin M. (2023). Photovoltaics at multi-terawatt scale: Waiting is not an option. Science.

[18] Anctil A., Beattie M.N., Case C., Chaudhary A., Chrysler B.D., Debije M.G., Essig S., Ferry D.K., Ferry V.E., Freitag M. (2023). Status report on emerging photovoltaics. J. Photonics Energy.

[19] Lloret T., Morales-Vidal M., Nieto-Rodríguez B., García-Vázquez J.C., Beléndez A., Pascual I. (2024). Building-Integrated Concentrating Photovoltaics based on a low-toxicity photopolymer. J. Phys. Energy.

[20] Morales-Vidal M., Lloret T., Ramírez M.G., Beléndez A., Pascual I. (2022). Green and wide acceptance angle solar concentrators. Opt. Express.

[21] Lloret T., Navarro-Fuster V., Ramírez M.G., Ortuño M., Neipp C., Beléndez A., Pascual I. (2018). Holographic lenses in an environment-friendly photopolymer. Polymers.

[22] Zhu L., Cui Y. Limit of resolution for holographic lenses. Proceedings of the Holography, Diffractive Optics, and Applications.

[23] Zhu L., Cui Y. Abnormal phenomena in resolution limit of holographic lens. Proceedings of the Applications of Photonic Technology 5.

[24] Lloret T., Navarro-Fuster V., Ramirez M., Morales-Vidal M., Beléndez A., Pascual I. (2020). Aberration-Based Quality Metrics in Holographic Lenses. Polymers.

[25] Yeom J., Jeong J., Hong J., Choi K.s. (2022). Analysis on image quality of a holographic lens with a non-converging signal wave for compact near-eye displays. Opt. Express.

[26] Lloret T., Morales-Vidal M., Navarro-Fuster V., Ramirez M., Beléndez A., Pascual I. (2022). Holographic Lens Resolution Using the Convolution Theorem. Polymers.

[27] Lloret T., Navarro-Fuster V., Morales-Vidal M., Ramírez M.G., Márquez A., Beléndez A., Pascual I. CCD and Hartmann-Shack wavefront sensor to analyse holographic lens resolution. Proceedings of the Holography: Advances and Modern Trends VIII.

[28] Calixto S., Alfaro-Gomez M. (2025). Dichromated Gelatin in Optics. Gels.

[29] Blanche P.A. (2020). Holographic recording media and devices. Optical Holography.

[30] Berramdane K., Lucío M.I., Ramírez M.G., Navarro-Fuster V., Bañuls M.J., Maquieira Á., Morales-Vidal M., Belendez A., Pascual I. (2024). Storage Optimization of Transmission Holographic Gratings in Photohydrogels. ACS Appl. Mater. Interfaces.

[31] Friedman M. (2003). Chemistry, Biochemistry, and Safety of Acrylamide. A Review. J. Agric. Food Chem..

[32] Ortuño M., Fernández E., Gallego S., Beléndez A., Pascual I. (2007). New photopolymer holographic recording material with sustainable design. Opt. Express.

[33] Ortuño M., Gallego S., Márquez A., Neipp C., Pascual I., Beléndez A. (2012). Biophotopol: A Sustainable Photopolymer for Holographic Data Storage Applications. Materials.

[34] Champagne E.B. (1967). Nonparaxial Imaging, Magnification, and Aberration Properties in Holography∗. J. Opt. Soc. Am..

[35] Latta J.N. (1971). Computer-Based Analysis of Hologram Imagery and Aberrations II: Aberrations Induced by a Wavelength Shift. Appl. Opt..

[36] Goodman J.W. (2017). Introduction to Fourier Optics.

[37] Mahajan V.N. (1991). Aberration Theory Made Simple.

[38] Dubois F., Schockaert C., Callens N., Yourassowsky C. (2006). Focus plane detection criteria in digital holography microscopy by amplitude analysis. Opt. Express.

[39] Gao P., Yao B., Rupp R., Min J., Guo R., Ma B., Zheng J., Lei M., Yan S., Dan D. (2012). Autofocusing based on wavelength dependence of diffraction in two-wavelength digital holographic microscopy. Opt. Lett..

[40] Hennelly B.M., Kelly D.P., Pandey N., Monaghan D.S. Review of Twin Reduction and Twin Removal Techniques in Holography. Proceedings of the China-Ireland Information and Communications Technologies Conference.

[41] Gerchberg R.W. (1972). A practical algorithm for the determination of phase from image and diffraction plane pictures. Optik.

[42] Perucho B., Micó V. (2014). Wavefront holoscopy: Application of digital in-line holography for the inspection of engraved marks in progressive addition lenses. J. Biomed. Opt..

[43] Vila-Andrés R., Esteve-Taboada J.J., Micó V. (2024). Soft Contact Lens Engraving Characterization by Wavefront Holoscopy. Sensors.

[44] Merchán Price M.S., Acosta Yepes N.F., Gonzales Rodríguez M.L., Cortés Rodríguez D.C. (2010). Snellen visual acuity versus spatial frequency of preferential looking test. Cienc. Tecnología Para Salud Vis. Ocul..

